# Vitamin D and the Kidney: Two Players, One Console

**DOI:** 10.3390/ijms23169135

**Published:** 2022-08-15

**Authors:** Fulvia Zappulo, Maria Cappuccilli, Alessandra Cingolani, Anna Scrivo, Anna Laura Croci Chiocchini, Miriam Di Nunzio, Chiara Donadei, Marianna Napoli, Francesco Tondolo, Giuseppe Cianciolo, Gaetano La Manna

**Affiliations:** Nephrology, Dialysis and Renal Transplant Unit, IRCCS-Azienda Ospedaliero-Universitaria di Bologna, Alma Mater Studiorum University of Bologna, 40138 Bologna, Italy

**Keywords:** calcium, cardiovascular disease, cholecalciferol, chronic kidney disease–mineral and bone disorder, fibroblast growth factor 23, kidney transplantation, parathyroid hormone, vitamin D, vitamin D receptor

## Abstract

Vitamin D belongs to the group of liposoluble steroids mainly involved in bone metabolism by modulating calcium and phosphorus absorption or reabsorption at various levels, as well as parathyroid hormone production. Recent evidence has shown the extra-bone effects of vitamin D, including glucose homeostasis, cardiovascular protection, and anti-inflammatory and antiproliferative effects. This narrative review provides an overall view of vitamin D’s role in different settings, with a special focus on chronic kidney disease and kidney transplant.

## 1. Introduction

The denomination “vitamin D” refers to a group of liposoluble, steroidal compounds crucial for intestinal absorption and for metabolism regulation of calcium and phosphates [1]. The most important isoforms in human physiology are ergocalciferol (vitamin D2) and cholecalciferol (vitamin D3), also known as calciols; while the first one is only synthesized in plants and fungi (dietary intake), the second one is both exogenous and produced endogenously from the photolysis of 7-dehydrocholesterol by UVB radiation in the skin [2]. Calciols undergo a two-step hydroxylation to turn into the biologically active form, calcitriol. First, vitamin D 25-hydroxylase in the liver mediates D2/D3 to change into 25(OH)D (calcidiol), a quantifiable form mostly used to determine vitamin D levels in serum, and it is defined as a native form. The next step is the hydroxylation on carbon 1 in the kidney’s proximal tubule to form calcitriol, also referred as 1,25-dihydroxyvitamin D [1,25(OH)2D]. Serum 1,25(OH)2D provides little information about vitamin D status, and it is usually normal or even elevated when hyperparathyroidism associates with vitamin D deficiency [3].

1,25(OH)2D reaches the target organs via a vitamin D-binding protein (VDBP) in systemic circulation, then binds to the local vitamin D receptor (VDR). It is known that the VDR belongs to a wide group of ligand-activated nuclear transcription factors, and it can boast an almost ubiquitous and tissue-dependent expression in nucleated cells [4]. Besides triggering absorption, output, and mobilization of both calcium and phosphorus, vitamin D also exerts several non-osteogenic and non-calcemic functions, thus representing a key player in extraskeletal health [3].

**Figure 1:**
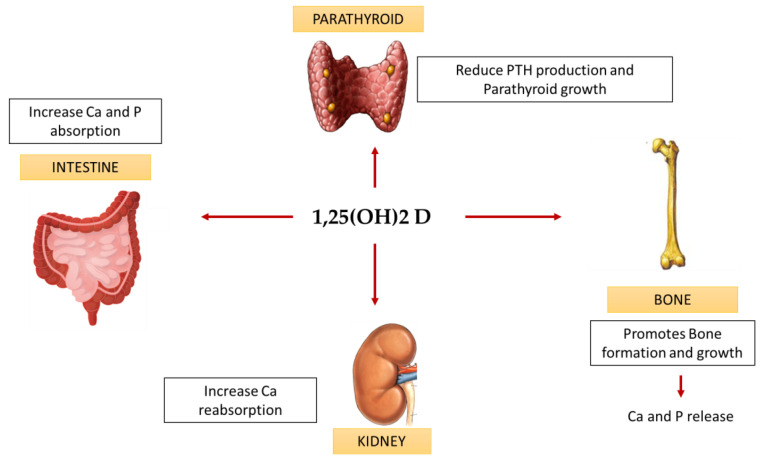
Systemic effect of vitamin D. Ca, calcium; P, phosphorus; PTH, parathyroid hormone.

To avoid intoxication, calcidiol and calcitriol are strictly regulated by 25(OH)D 24-hydroxylase (CYP24A1), which is the primary vitamin D-inactivating enzyme for both compounds [5]. Moreover, the parathyroid hormone (PTH) and fibroblast growth factor 23 (FGF23) also regulate vitamin D metabolism. PTH is produced by the parathyroid glands secondarily to low serum calcium levels; it both stimulates bone turnover and upregulates 1,25(OH)2D levels due to the induction of renal expression of the involved cytochrome (CYP27B1). FGF23 instead is produced by osteoblasts and osteoclasts in response to high phosphate and calcitriol serum levels and downregulates calcitriol production by inhibiting CYP27B1 in the kidney [6,7]. In Figure 1, the main systemic effect of 1,25(OH)2D are exposed.

## 2. Vitamin D in Bone Homeostasis

Vitamin D has direct and indirect control of bone-matrix formation, as its main physiologic function is the modulation of calcium and phosphorus absorption or reabsorption at various levels. In this frame, the kidney has a major involvement: once calcium and inorganic phosphorus are filtered to preurine, 1,25(OH)2D, together with PTH, regulates their reabsorption through various channels and transporters in distal, tubular segments [8]. In conditions of normal renal function, about 98% of the filtered calcium is reabsorbed in the kidney; in proximal tubules, where thiazide diuretics, 1,25(OH)2D, and PTH have no influence, Na-dependent, paracellular mechanisms mediate the uptake of 50–60% of the whole load of calcium. The descending loop and the thin, ascending limb of the loop of Henle play only a minor role in calcium homeostasis. On the other hand, important percentages of the reuptake of the filtered mineral occur in the thick, ascending limb (20%), distal tubule (10–15%), and collecting duct (5%), where calcium reabsorption is ATP dependent and mediated by epithelial calcium channels, calbindin, and the plasma membrane Ca^2+^ ATPase (ATP2B1) [9,10,11].

Another important function of vitamin D is the enhancement of intestinal calcium and phosphorus reabsorption. This is indeed demonstrated by the great vitamin D influence on the amount of enteric calcium uptake: with 25(OH)D insufficiency, only 10–20% of dietary calcium intake eventually enters into the bloodstream, while adequate levels of the prohormone improve the absorption to 30–40% [12,13]. Many of the direct effects of vitamin D on the skeletal tissue are not completely known. However, there is a large amount of evidence to suggest that vitamin D involvement in bone-tissue deposition and remodeling is represented not only through the regulation of Ca/P serum levels with the close coordination of PTH, but also via the direct effect on bone cells expressing VDR, osteoblasts, and osteoclasts [14]. Despite the fact that the 1α-hydroxylation of 25(OH)D to 1,25(OH)_2_D in bone cells was described many years ago, the discovery of its autocrine/paracrine activity for osteoblast and osteoclast maturation and proliferation is relatively recent [15]. It has been proven that 1,25(OH)_2_D promotes the expression of RANKL, osteocalcin, and osteopontin, associated with osteoblast maturation and mineralization. Moreover, 1,25(OH)2D also controls hyperactive osteoclastic resorptive activity and upregulates the expression of FGF23 and sclerostin via the VDR [16].

## 3. Vitamin D in Chronic Kidney Disease and End Stage Renal Disease

Patients with chronic kidney disease (CKD) and end-stage renal disease (ESRD) present more severe vitamin D deficiency and insufficiency compared to the healthy population. Different definitions of vitamin D deficiency and insufficiency have been provided over the last years, resulting in heterogeneous guidelines, ranges, and cut-offs. However, most clinicians refer to the Endocrine Society’s recommendations, where 25(OH)D concentrations < 20 ng/mL are defined as deficiency, concentrations between 21 and 29 ng/mL as insufficiency, and serum levels > 30 ng/mL as normal/sufficiency [16]. Given the serious dietary restrictions in subjects with impaired renal function and the presence of comorbidities that may influence hospitalization and mobility (leading to lower sun exposure), CKD patients commonly require vitamin D supplementation, mainly cholecalciferol and calcifediol-based supplements [17]. Moreover, the 1α-hydroxylation of 25(OH)D is impaired due to damaged kidney tissue. The resulting hypocalcemia and hyperphosphoremia, secondary to kidney failure, lead to secondary hyperparathyroidism and increased serum levels of the hyperphosphaturic, osteocyte-derived fibroblast growth factor 23 (FGF23) [18]. PTH and FGF23 have opposite effects on the regulation of 1α-hydroxylase: while PTH enhances its expression in order to invert the trend of calcium loss, FGF23, which is triggered by phosphate retention, inhibits renal 1α-hydroxylase expression [7]. Long-term 25(OH)D and 1,25(OH)2D insufficiency and secondary hyperparathyroidism result in a broad spectrum of bone damage, commonly found in the CKD/ESRD population known as chronic kidney disease–mineral and bone disorder (CKD-MBD) [19].

## 4. Vitamin D and CKD-MBD

Protracted 25(OH)D and 1,25(OH)2D deficiency causes a drop in bone mineral density and progressive bone loss, thus burdening the patient with a wide range of bone disorders, a higher risk of pathological fractures, significant morbidity and mortality, and ultimately, increased healthcare costs [20,21].

In clinical practice, multiple designations are used to indicate CKD-related bone diseases, and they can be summed up in three fundamental, pathological entities: osteoporosis, CKD-MBD, and renal osteodystrophy [22].

Osteoporosis is defined as a systemic, skeletal disorder, where bone strength and resistance are compromised, and thus, affected patients have an elevated risk of fracture due to reduction in bone mass density (BMD, mineral quantity per square centimeter, expressed as g/cm^2^) and bone quality (BQ, comprehensive of microarchitecture, mineralization, turnover, and microcrack accumulation) [23,24,25,26,27,28]. According to the World Health Organization (WHO), “osteoporosis is defined as a BMD that lies 2.5 standard deviations or more below the average value for young healthy women (a T-score of < −2.5 SD)”. A second, higher threshold that lies between −1 and −2.5 SD describes “low bone mass” or osteopenia [23].

**Figure 2:**
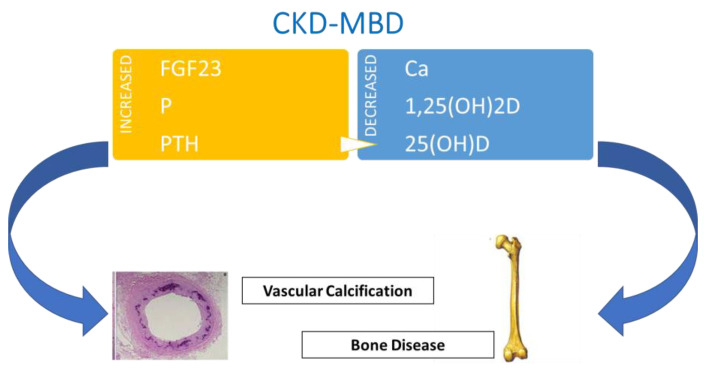
CKD-MBD pathogenesis and its main systemic effects. FGF23, fibroblast growth factor 23; P, phosphorus; PTH, parathyroid hormone; Ca, calcium.

CKD-MBD is a systemic disorder of mineral metabolism, initiated by phosphorus retention and elevated levels of FGF23 and PTH, resulting in a detrimental rebound on skeletal integrity. The disease is characterized by alterations of the principal CKD-MBD biomarkers (calcium, phosphorus, vitamin D, and PTH) associated with anomalies in bone turnover, mineralization, and volume (TMV); extraskeletal calcifications; and atherosclerosis [29]. In Figure 2, pathogenesis of CKD-MBD is schematized. 

Lastly, the denomination “renal osteodystrophy” describes the different morphological pictures of bone disease that can be diagnosed in CKD through bone biopsy [30], according to TMV classification. In this case, the cortical bone is of predominant interest [22]. Osteitis fibrosa cystica is the main one among these skeletal disorders and is characterized by high bone turnover that triggers the production of fibrous bone instead of resistant, lamellar bone, resulting from high serum PTH levels [22]. Conversely, in adynamic bone disease, low bone turnover is common, due to reduced osteoblasts and osteoclasts activity. The ability of bone to release or store calcium is consequently compromised, resulting in broad oscillation of calcium levels [24,25].

The physiological concentration of 25(OH)D has inhibitory effects on PTH transcription [28]. In secondary hyperparathyroidism, 25(OH)D has a synergistic effect with 1,25(OH)2D on PTH production [28]. 

Vitamin D deficiency (both 25(OH)D and 1,25(OH)2D) is highly prevalent in the CKD population. Previously, a cross-sectional analysis of 825 HD patients showed that 78% of the cohort had vitamin D (25(OH)D) deficiency (<30 ng/mL) and 18% had severe deficiency (<10 ng/mL). Moreover, they demonstrated that 25(OH)D deficiency was associated with increased early mortality [28]. This phenomenon contributes to the development of high PTH levels and the worsening of secondary hyperparathyroidism.

Some studies have reported the association between free 25(OH)D and serum PTH decline [31]. Nevertheless, some others have not reached such conclusions [32]. In fact, it is still uncertain if levels of 25(OH)D may represent the total, biologically active vitamin D. In fact, supplements of both cholecalciferol and calcifediol are effective in increasing the total and free 25(OH)D level and are associated with a serum PTH-level decline [33]. In CKD patients, supplementation with cholecalciferol showed a significant increase in serum 25(OH)D concentration and a decrease in PTH levels when compared with the placebo [34]. More recently, Westerberg reported that high-dose cholecalciferol (8000 IU/day) in patients with CKD stages 3–4 prevents the development of secondary hyperparathyroidism, with no increase in the risk of hypercalcemia and hyperphosphatemia [35].

The 2017 KDIGO CKD-MBD Guideline suggests that vitamin D deficiency should be corrected if CKD stages 3 to 5a, not-yet-dialyzed-patients have a progressive or persistently high PTH level [19]. Vitamin D administration can be considered the adjuvant therapy for secondary hyperparathyroidism prevention because of the high prevalence of vitamin D deficiency in the general population and in CKD patients. Moreover, vitamin D has multiple pleiotropic and systemic effects, as described above. Although more evidences support the benefit of initiating vitamin D supplementation to lower the development of secondary hyperparathyroidism, the efficiency of vitamin D administration for this purpose still needs more randomized, controlled trials to prove. 

## 5. Effect of Vitamin D Therapy

Due to the long life of complex 25(OH)D and the vitamin D-binding protein (15 days), daily, weekly, or monthly administration regimens can be efficient for restoring 25(OH)D levels [18,36,37].

At present, there is no current evidence to prefer one formulation of nutritional vitamin D over another in CKD, and no evidence has been found analyzing the benefit that derives from combining nutritional (ergocalciferol, cholecalciferol, and calcifediol) and activated vitamin D (VDRAs, calcitriol, and paricalcitol) [14]. The latest reports indicate that in patients with CKD, nutritional forms of vitamin D have poor PTH-lowering efficacy and vitamin D supplementation is inferior to VDRAs for hyperparathyroidism treatment, particularly in dialysis patients [14,27]. However, cholecalciferol supplementation in dialysis patients causes an increase in both 25(OH)D and 1,25(OH)2D levels, suggesting that extra-renal activity may be significant in these patients [14]. These effects depend on the vitamin D dosage, the type of vitamin D compounds, the duration of the study, and the examined population. 

Kandula et al. reported that nutritional vitamin D leads to increased 25(OH)D levels without influencing calcium and phosphorus levels but causes a reduction in the serum PTH level (41% decrease), mostly in dialysis patients [38,39,40]. Jean et al. described a positive effect of systematic 25(OH)D supplementation during the pre-dialysis period to prevent secondary hyperparathyroidism (SHPT) [41]. 

Concerning the mineral metabolism, vitamin D has shown multiple effects that involve renal failure progression and cardiovascular disease. High-dose cholecalciferol administration seems to ameliorate cardiovascular and endothelial parameters in children with CKD, measured through flow-mediated dilatation, arterial stiffness, and plasmatic dosage of homocysteine and von Willebrand [42]. Nonetheless, Karakas et al. confirmed that the administration of cholecalciferol improved the percentage of flow-mediated dilatation in patients under chronic dialysis treatment [43]. 

In diabetic CKD patients using angiotensin-converting enzyme inhibitors, a decrease in proteinuria by adding native vitamin D was described [44]. A RCT by Meireless et al. revealed that cholecalciferol promoted the upregulation of CYP27B1 and VDR expression in monocytes and decreased serum IL-6 and C-reactive protein levels [45]. In a recent meta-analysis, Mann et al. lacked finding significant effects of vitamin D supplementation on mortality [46]. 

In 2014, a Cochrane analysis showed some evidences that vitamin D may decrease all-cause mortality and cancer mortality in elderly participants. Elevated urinary calcium excretion, renal insufficiency, cancer, and cardiovascular, gastrointestinal, psychiatric, or skin disorders were not statistically significantly influenced by vitamin D supplementation [47]. 

## 6. Vitamin D and Kidney Transplantation

In kidney transplant recipients, the underlying causes of the altered metabolism of vitamin D, referred to as both 25(OH)D deficiency and reduced levels of 1,25(OH)2D, are still unclear. Although many uremic alterations are recovered by the restored kidney function, vitamin D metabolism usually remains imbalanced and suboptimal [48]. 

As observed in CKD/ESRD patients, vitamin D deficiency represents a trigger of CKD-MBD, and it has been associated with worse clinical outcomes due to the impairment of its pleiotropic effects, especially those involving the renal and cardiovascular systems [16,37,43]. Vitamin D deficiency is associated with deteriorated kidney function and worse long-term clinical outcomes [49] that can be due to the higher rates of rejection episodes and proteinuria onset [50]. Filipov et al. demonstrated that poor vitamin D status results in higher proteinuria after kidney transplantation [51]. The possible antiproteinuric mechanisms of vitamin D are the inhibition of the renin–angiotensin–aldosterone system (RAAS), nuclear factor κΒ (NFKB1) inactivation, Wnt/β catenin (WNT1/CTNNB1) pathway suppression, and upregulation of slit-diaphragm proteins. However, up to now, there is not strong evidence of a favorable effect of vitamin D therapy as a disease-modifying factor in terms of proteinuria, interstitial fibrosis/tubular atrophy (IF/TA), or graft function [48,52].

Lifelong immunosuppressive therapy is mandatory in kidney transplants to prevent allograft rejection, and it might be one of the culprits of CKD-MBD: many studies have demonstrated how calcineurin inhibitors and steroids have a negative effect on the vitamin D system and bone metabolism [53], while sirolimus has been described as a bone-sparing drug, with no skeletal side effects [54].

Table 1 summarizes the main studies on the effects of 25(OH)D supplementation in renal patients. 

## 7. Immunomodulatory Effects of Vitamin D

The classic functions of vitamin D are the regulation of calcium in bone and mineral homeostasis [55]. In addition, VDR is expressed in immune cells, such as macrophages, dendritic cells, B and T lymphocytes, and neutrophils. This suggests that vitamin D may play an important role in the regulation of the immune system [56,57].

Recently, some studies have shown that 1,25(OH)2D regulates both adaptive and innate immunity but in opposite directions. In fact, 1,25(OH)2D inhibits the adaptive immune response and enhances the innate immune response [58]. 

Previously, some studies have demonstrated vitamin D-dependent, antimicrobial activity [59]. In particular, calcitriol can reduce the expression of MHC class II molecules, as well as co-stimulatory molecules (CD80, CD86), which also results in a decline of IL-12 secretion [60].

Chen et al. studied the effect of 25(OH)D administration on innate immune cells. They found an enhanced production of IL-1beta and IL-8 by both neutrophils and macrophages, while the phagocytic capacity was suppressed in these cells [61]. 

Furthermore, the immune-modulating effects of vitamin D and its analogs have been well-characterized in dendritic cells: these cells are antigen-presenting cells that stimulate lymphocytes through antigen presentation. Griffin et al., have shown a robust vitamin D-dependent inhibition of the maturation, differentiation, and survival of dentritics cells [62]. Moreover, in the course of the inflammatory process, vitamin D strongly inhibits the migration and maturation of dentritics cells, causing a reduction in antigen presentation and an activation of T cells. Furthermore, Il-2 production decreases while IL-10 expression increases, leading to the suppression of the T helper 1 (Th1) phenotype. Therefore, by maintaining dentritic cells in an immature phenotype, vitamin D and its analogs contribute to an induction of a tolerogenic state [63,64]. In addition, vitamin D suppresses the proliferation of B cells and immunoglobulin production. It also suppresses the differentiation of B cells into plasma cells [65,66]. Naïve B cells express very low levels of VDR. However, the activation of B cells induces VDR expression. Moreover, vitamin D signaling potentiates apoptosis of activated B cells and inhibits memory B-cell formation and the secretion of immunoglobulins IgG and IgM in activated B cells [67].

## 8. Pleiotropic Effects of Vitamin D

Over the last few years, increasing evidence has been revealed about the impact of vitamin D on cardiovascular health, inflammatory status, cancer, and progression of CKD. The discovery of the VDR enabled multiple investigations on the association of vitamin D deficiency with acute and chronic diseases. Due to the wider distribution of the VDR, vitamin D is associated with several pleiotropic effects: renal-function preservation, regulation of blood pressure, glycemic control, regulation of cellular proliferation, regulation of the renin-angiotensin-aldosterone system (RAAS), and immunomodulation properties [68,69].

Vitamin D plays a central role in cardiovascular health, as shown by the expression of the dedicated signaling apparatus at almost all levels of the cardiovascular system, i.e., endothelial cells, cardiomyocytes, and smooth muscle cells of vessels [70,71,72,73]. Experimental studies conducted on VDR-knockout mice highlighted a dramatic increase in cardiovascular dysfunction in affected animals that developed ventricular hypertrophy, heart failure, hypertension, and upregulation of RAAS. Evidence suggests that such comorbidities improve following vitamin D supplementation [4].

It has been found that 25(OH)D deficiency is associated with accelerated arteriosclerosis and endothelial dysfunction in ESRD patients, with a subsequent increase in cardiovascular risk. Moreover, a suppression of cardiomyocytes proliferation in case of vitamin D deficiency has been hypothesized [74].

Several prospective observational studies investigated 25(OH)D levels and the risk of CVD, and the clinical endpoints were various myocardial infarction, combined cardiovascular disease, stroke, and cardiovascular mortality [75]. The Framingham Offspring Study recruited 1739 participants free of CVD at the baseline. Over an average follow-up time of 5 years, lower 25(OH)D levels were associated with a risk of cardiovascular events that was 1.62 times higher [72]. Similarly, the Health Professionals Follow-up Study revealed that the incidence of acute myocardial infarction was 2.42 times higher in men with 25(OH)D levels < 15 ng/mL, compared to those with levels above 30 ng/mL [76]. On the other hand, the NHANES III study, which included data from more than 13,300 participants followed for 8.7 years, showed only a trend towards an increased risk in the lowest (<17.8 ng/mL) compared with the highest 1,25(OH)2D [77]. In a prospective cohort study, as the subset of the MrOS study, no significant association was found between 25(OH)D deficiency (<15 ng/mL) and cardiovascular incidence (coronary heart disease and cerebrovascular attack) compared with vitamin D sufficiency (>30 ng/mL) [78].

Several studies evaluated not only have changes in cardiovascular risk with low 25(OH)D levels, but also with the contribution of higher levels. Most of these suggest that risk does not decrease with levels >30 ng/mL [79,80]. Some others even suggested a possible U-shaped relation, with a possible increase in cardiovascular disease risk at high 25(OH)D D levels (>60 ng/mL) [81]. Finally, if the observational data provided evidence of the association between low 25(OH)D levels and increased cardiovascular risk, evidences are still limited to support the view that higher levels of 25(OH)D are linked with a similar decrease in risk.

Regarding the control of the inflammatory status, accumulating data indicate that vitamin D exerts anti-inflammatory effects through many ways, namely by inhibition of the prostaglandin pathway, proinflammatory cytokines, and NFKB. Moreover, it provides antioxidant defense against ROS, thus avoiding the perpetuation of pro-inflammatory responses and DNA damage [82].

Another function attributed to vitamin D is the ability to promote the differentiation of monocytes into macrophages, lymphocytes, and dendritic cells, which are the first line of defense of the innate immune system and infection control [83].

Several studies have also highlighted an association between sufficient vitamin D status and cancer prevention in several malignancies, namely prostate, breast, and colon cancer. This protective role can be explained by vitamin D-mediated upregulation of the cyclin-dependent kinase inhibitors p21 and p27 and inhibition of the TGF-α/EGFR growth pathway [84].

**Figure 3:**
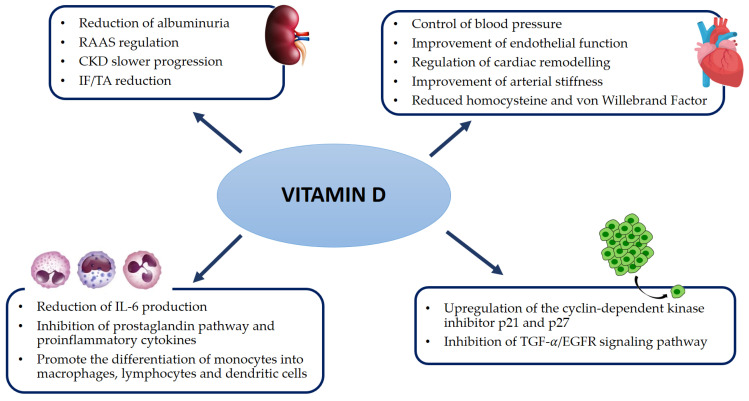
Pleiotropic effect of vitamin D. CKD, chronic kidney disease; EGFR, epidermal growth factor receptor; ESRD, end-stage renal disease; IF/TA, interstitial fibrosis/tubular atrophy; IL-6, interleukin 6; RAAS, renin-angiotensin-aldosterone system; TGF-α, transforming growth factor alpha.

Furthermore, many studies focused on nephropathies reported that active vitamin D protects the kidneys through its anti-inflammatory and antifibrotic effects. Calcitriol has proven to have inhibitory effects on renal interstitial myofibroblasts, thus decelerating the progression to renal interstitial fibrosis. Experimental studies involving knockout mice lacking active vitamin D receptors revealed elevated levels of renin and angiotensin II in the mice’s blood, which caused a significant rise in blood pressure and subsequent cardiac hypertrophy [85,86,87,88]. Figure 3 is a schematic representation of the main pleiotropic systemic effects of vitamin D.

## 9. Conclusions

Recently, the function of vitamin D has been extensively investigated. The discovery of the VDR can lead to a better understanding of the relationship of acute and chronic diseases with vitamin D deficiency. Results of vitamin D trials vary for the general population and renal patients. The discrepancies may be due to differences in the baseline serum 25(OH)D levels, vitamin D doses and treatment periods, adherence to supplementation, and VDR genetic polymorphisms [89].

Therefore, the application of vitamin D in disease treatment and prevention is far from been achieved. Further investigation is required to pursue this aim. Regarding vitamin D reference values, there is so far still no univocal consensus on the reference values of vitamin D’s status. The optimal serum concentration of 25(OH)D has been considered to not lead to a PTH elevation [90]. Such a view seems to be obsolete, and it is the result of partial knowledge of the biological activity of vitamin D. Moreover, the bioaccessibility of vitamin D in foods must be considered. There is, however, a lack of kinetic data that allows for the prediction of vitamin D’s stability under industrial processing conditions [91]. 

## Figures and Tables

**Table 1:** Most representative studies on the effects of native vitamin D supplementation in the nephrology clinical setting.

Authors	Vitamin D Formulation	Dosage	Study Design	Patients	Study Length	Results
Kandula et al. [38]	Ergocalciferol or cholecalciferol	Observational study 4000 to 50,000 IU daily. RCTs rom 20,000 IU weekly to 25,000 IU monthly	Systematic review and meta-analysis	CKD: pre-dialysis, hemodialysis, peritoneal dialysis and KTRs	1966 to September 2009	No influence on Ca and P levels Reduction of PTH
Alvarez et al. [39]	Cholecalciferol	50,000 IU/week for 12 weeks followed by 50,000 IU every other week for 40 weeks	Prospective	46 early CKD (stages 2–3)	1 year	Prevent vitamin D insufficiency Improvement of serum PTH
Cupisti et al. [40]	Cholecalciferol	10,000 IU once-a-week	Cohort study	405 CKD patients (stages 2–4)	12 months	Reduction of PTH
Jean et al. [41]	Cholecalciferol and calcifediol	cholecalciferol 100,000 U/month calcifediol 10–50 μg/d	Prospective	All incident and prevalent hemodialysis patients in a single center	Three observation periods of 1-yr each	Reduction of the incidence of SHPT
Aytac et al. [42]	Cholecalciferol	single dose of 300,000 IU of oral cholecalciferol	Prospective	41 CKD children and 24 healthy subjects free of any underlying cardiac or renal disease	12 weeks	Increase in flow mediated dilatation, reduction in arterial stiffness Reduction of plasmatic Hcy and von Willebrand factor
Karakas et al. [43]	Cholecalciferol	50,000 units weekly	Prospective	44 hemodialysis patients and 24 healthy	8 weeks	Increase in flow-mediated dilatation
Kim et al. [44]	Cholecalciferol	40,000 units weekly for 8 weeks and then monthly	Prospective	63 patients with diabetic nephropathy	4 Months	Decrease in proteinuria in addition to ACE-i
Meireless et al. [45]	Cholecalciferol	50,000 IU of cholecalciferol twice weekly	Prospective	38 dialysis patients	12 weeks	Upregulation of CYP27B1 and VDR expression in monocytes Lower serum IL-6 and CRP levels
Mann et al. [46]	Cholecalciferol, doxecalciferol, paracalcitol or alfacalcidol	0.25 ug per day to 200,000 IU per week	Systematic review	Adults with CKD (≤60 mL/min/1.73 m^2^), including dialysis-dependent ESRD	3–104 weeks	Lack of significant effects of vitamin D supplementation on mortality

ACE-I, angiotensin-converting enzyme inhibitors; CKD, chronic kidney disease; CRP, C-reactive protein; ESRD, end stage renal disease; IL-6, interleukin 6; Hcy, homocysteine; PTH, parathyroid hormone; SHPT, secondary hyperparathyroidism; VDR, vitamin D receptor.

## Data Availability

Not applicable.
